# Constructing national identity: media narratives on the naturalization of football players in Indonesia

**DOI:** 10.3389/fspor.2025.1595501

**Published:** 2025-07-15

**Authors:** Ahmad Ismail, Hardiyanti Munsi, Semiarto Aji Purwanto

**Affiliations:** ^1^ Department of Anthropology, Hasanuddin University, Makassar, Indonesia; ^2^ Department of Anthropology, University of Indonesia, Depok, Indonesia

**Keywords:** media narratives, naturalization in football, national identity, sports globalization, Indonesia

## Abstract

This study explores how Indonesian media construct narratives surrounding the naturalization of foreign-born football players and their implications for national identity. Drawing on 52 media articles from major national outlets published between January 2022 and April 2025, the research employs a qualitative content analysis grounded in a multi-theoretical framework that includes imagined communities (Anderson), identity representation (Hall), and thick vs. thin citizenship (Oonk). The findings reveal three dominant media frames: (1) naturalized players as national assets who elevate performance and international competitiveness; (2) skepticism over their authenticity and emotional ties to Indonesia; and (3) contested implications for local talent development. While naturalization is often portrayed as a pragmatic strategy, the media also frame it as a site of negotiation between globalization and local values. By mediating public perception, the media significantly influence how naturalized players are integrated into the symbolic boundaries of national identity. This study contributes to scholarship on media, sports, and nationalism in the Global South by showing how national identity is constructed through media discourses in the era of sports globalization.

## 1 Introduction

The phenomenon of naturalization in sports has become a prominent global issue, reflecting the complex interplay between globalization, identity, and national representation (1–4). Naturalized athletes, particularly in football, often contribute significantly to the success of their adopted countries, bringing a blend of talent and diversity to the teams they represent (4). According to FIFA's 2022 report, over 10% of players in major international tournaments are naturalized citizens, highlighting the growing reliance on foreign-born athletes to achieve competitive advantage. Scholars such as (5, 6) argue that naturalization is a double-edged sword: while it enhances athletic performance, it also raises debates around national identity and the authenticity of representation in sports. This duality underscores the need to examine how societies perceive and construct narratives around naturalized athletes.

In recent years, under the guidance of coach Shin Tae-yong, the Indonesian national football team has actively pursued the naturalization of foreign-born players to enhance its competitive edge. Notable additions include Maarten Paes, a goalkeeper for FC Dallas in Major League Soccer, who became an Indonesian citizen on April 30, 2024. Similarly, Mees Hilgers, a defender for FC Twente in the Netherlands, joined the national team in September 2024. Other significant naturalized players are Shayne Pattynama, Jordi Amat, Justin Hubner, Sandy Walsh, and Calvin Verdonk, each bringing valuable international experience to the squad. This strategy has sparked diverse reactions among the Indonesian public and media. While some view it as a pragmatic approach to elevate the national team's performance, others express concerns regarding the implications for local player development and national identity. Coach Shin Tae-yong himself has acknowledged the benefits of naturalized players, stating that their presence adds strength and depth to the team. However, he also emphasizes the importance of local players striving to improve, ensuring healthy competition within the squad.

Existing literature has extensively explored the role of media in shaping public perception of naturalized athletes (7–13). Studies by (13, 14) highlight how media framing influences the integration and acceptance of naturalized players within their host nations. However, these studies primarily focus on Western contexts, leaving a gap in understanding how non-Western countries like Indonesia engage with this phenomenon. Furthermore, while previous research has examined the intersection of sports and nationalism, it often overlooks the nuanced role of local media in constructing narratives around naturalization.

This article seeks to address these gaps by analyzing how Indonesian media frame the naturalization of football players and its implications for national identity construction. By focusing on a non-Western context, this study expands the existing body of knowledge and offers a localized perspective on the global phenomenon of naturalization in sports. The primary objective of this research is to explore the themes, patterns, and contradictions within media narratives and to assess their impact on public perceptions of national identity.

This article seeks to address these gaps by analyzing how Indonesian media frame the naturalization of football players and its implications for national identity construction. Specifically, this research aims to answer two research questions:

• RQ1: How do Indonesian media frame the naturalization of football players in terms of their contribution to national identity and team performance?
• RQ2: What are the dominant themes, patterns, and contradictions in media narratives regarding the naturalization of foreign-born football players in Indonesia?

The central argument of this article is that Indonesian media play a pivotal role in shaping the duality of pride and skepticism surrounding naturalized football players. This study hypothesizes that media framing not only reflects societal tensions but also actively constructs and negotiates the boundaries of national identity in a globalized era. Through qualitative content analysis, this research aims to provide a comprehensive understanding of how media narratives influence identity and belonging in the context of Indonesian football. This study offers a critical theoretical contribution by situating the phenomenon of athlete naturalization within the broader discourse of national identity construction in the Global South, specifically Indonesia. Unlike much of the existing literature which predominantly focuses on Western contexts, this paper foregrounds how postcolonial nations negotiate identity, belonging, and legitimacy through media portrayals of foreign-born athletes.

Building on Anderson's concept of *imagined communities* (15), this research argues that media narratives function as powerful instruments that extend and reshape the symbolic boundaries of national membership. Naturalized football players, through repeated representation in mainstream media, are framed either as authentic members of the national community or as outsiders, reflecting a tension between pragmatic nationalism and cultural essentialism. Moreover, the study draws on the concept of “media framing as identity negotiation”, where framing is not merely a reflection of public sentiment but an active process in defining who belongs and under what terms. This approach expands the theoretical discussion on sport-mediated citizenship by integrating notions of emotional attachment, performative nationalism, and mediated authenticity.

In doing so, this paper contributes to the theoretical intersection of media anthropology, nationalism studies, and sports globalization by highlighting how identity is not only contested at the level of state institutions or grassroots movements but also in the everyday discursive practices of sports journalism. It further challenges the assumption that naturalization is a purely administrative or strategic choice, proposing instead that it is a contested site of national meaning-making shaped by both global pressures and local values.

## 2 Theoretical framework

The naturalization of foreign-born athletes, particularly in football, has emerged as a critical arena in which questions of national identity, citizenship, and belonging are negotiated—both in policy and in media discourse. Previous studies have explored this phenomenon within the broader context of globalization and nationalism, emphasizing the symbolic function of sport as a site for performing collective identity (2, 14, 16). However, much of this literature focuses on Western or Global North contexts, leaving a substantial gap in understanding how Global South nations like Indonesia interpret and contest naturalization as a cultural and political act.

To bridge this gap, this study draws upon a triangulated theoretical framework grounded in nationalism studies, cultural representation, and the sociology of sport. At its core lies Benedict Anderson's (15) concept of *imagined communities*, which views the nation as a socially constructed entity, made real through shared narratives, rituals, and media. In the Indonesian case, the national football team—often referred to as *Timnas Garuda*—serves as a key platform for articulating this imagined community. Naturalized athletes who were not born, raised, or linguistically aligned with Indonesia challenge the conventional boundaries of this national imagination. Their inclusion prompts a reevaluation of who can be considered a legitimate member of the nation.

Adding to this, Stuart Hall's (17) theory of identity as a representational process provides insight into how media acts as a central mechanism in the construction of belonging and otherness. Hall argues that identity is not fixed but formed through cultural positioning and discursive practices. Media representations of naturalized athletes—whether celebratory, skeptical, or ambivalent—construct a spectrum of inclusion, framing them either as national heroes or as outsiders occupying privileged sporting roles. In this framework, football becomes a “cultural battlefield” where notions of *Indonesian-ness* are actively contested, often under the guise of sports journalism.

Further, the work of Gijsbert Oonk (1) introduces a crucial distinction between “thin” and “thick” forms of citizenship. “Thin citizenship” refers to the legal and bureaucratic recognition of nationality, while “thick citizenship” encompasses cultural intimacy, emotional investment, and public legitimacy. The Indonesian public often evaluates naturalized athletes through this lens—questioning whether legal status alone suffices for full national acceptance, especially when players lack visible ties to Indonesian language, heritage, or community involvement.

This theoretical scaffolding is reinforced by recent empirical studies. Chaeroni (18), through a systematic literature review, demonstrated how sports serve as a vessel for the performance of nationalism and patriotism across diverse contexts. Their findings emphasize that while sports can unite a nation, they also reveal internal fractures over who counts as a legitimate symbol of national pride. Similarly, Cochrane, Amin, and Al-Kaabi (19) examined national identity construction in Qatar and found that media plays a central role in filtering which naturalized athletes are deemed culturally compatible with state-led identity narratives. Both cases parallel Indonesia, where the naturalization of players like Marc Klok or Shayne Pattynama is framed not just in terms of merit but also cultural resonance.

Taken together, these perspectives underpin the present study's core argument: that Indonesian media do not merely reflect public opinion on naturalized footballers—they actively shape it. Through thematic framing, the media constructs moral boundaries around inclusion, loyalty, and authenticity, thus contributing to the ongoing redefinition of what it means to be “Indonesian” in the age of sports globalization.

## 3 Method

The focus of this study was chosen to address the growing significance of naturalization in Indonesian football and its implications for national identity. Given the prominent role of media in shaping public opinion (20, 21), this research specifically examines how media narratives construct and negotiate the identity of naturalized football players. This focus aligns with the broader objective of understanding the relationship between media, nationalism, and globalization in the Indonesian context.

### 3.1 Data sources and selection criteria

This study utilizes qualitative content analysis of media texts as its primary methodological approach, drawing upon secondary data from prominent Indonesian news outlets. These media sources were selected for their national reach, editorial influence, and consistent coverage of sports and national identity issues (22).

The core data corpus comprises 52 media articles published between January 2022 and April 2025**,** retrieved from Kompas.com, DetikSport, CNN Indonesia, Tempo.co, Republika.co.id**,** and supplemented by selected opinion columns and press releases from PSSI *(Persatuan Sepakbola Seluruh Indonesia)**.*** These platforms were chosen for several reasons:

• National Circulation and Readership**:** These outlets represent both mainstream (e.g., Kompas, CNN Indonesia) and critical (e.g., Tempo, Republika) editorial perspectives.
• Topical Relevance**:** They regularly report on issues related to football, naturalization policies, and debates around national identity.
• Diversity of Framing Styles**:** These media sources offer a spectrum of narratives—from celebratory portrayals of naturalized players to skeptical commentaries on authenticity and cultural attachment.

A purposive sampling strategy was employed, using the keywords: *naturalisasi pemain*, *Timnas Indonesia*, *identitas nasional*, and *PSSI*. Articles were included if they:

• Contain direct reference to naturalized football players in Indonesia;
• Discuss issues of nationalism, citizenship, or belonging;
• Were published in credible, editorially-reviewed outlets.

Articles were excluded if they:

• Solely reported match results without commentary on identity or naturalization;
• Were redundant (e.g., syndicated duplicates across platforms);
• Contained insufficient narrative framing or lacked relevance to the research questions.

To enhance transparency and replicability, Table 1 includes a full list of the media articles analyzed, complete with title, outlet, publication date, and URL.

**Table 1:** List of analyzed media articles.

No	Article Title	Media Outlet	Date	Url
1	Timnas Indonesia Dipastikan Tanpa Pemain Naturalisasi Baru di Piala Asia 2025	Kompas.com	20-Apr-25	https://www.kompas.com/sulawesi-selatan/read/2025/04/20/102707388/timnas-indonesia-dipastikan-tanpa-pemain-naturalisasi-baru-di?page=all
2	Timnas Indonesia Dipastikan Tanpa Pemain Naturalisasi Baru di Sisa Babak Ketiga Kualifikasi Piala Dunia 2026	Kompas.com	20-Apr-25	https://www.kompas.com/sulawesi-selatan/read/2025/04/20/102707388/timnas-indonesia-dipastikan-tanpa-pemain-naturalisasi-baru-di?page=all
3	Yakin Menang Lawan Timnas Indonesia, Pelatih Bahrain Sindir Soal Pemain Naturalisasi di Skuad Garuda	Tempo.co	24-Mar-25	https://www.tempo.co/sepakbola/yakin-menang-lawan-timnas-indonesia-pelatih-bahrain-sindir-soal-pemain-naturalisasi-di-skuad-garuda-1223710
4	Kritik Tajam Pelatih Belanda: Sensasi Berlebihan Pemain Naturalisasi Timnas Indonesia	Kompas.com	22-Mar-25	https://www.kompas.com/jawa-barat/read/2025/03/22/164614588/kritik-tajam-pelatih-belanda-sensasi-berlebihan-pemain-naturalisasi
5	Kualifikasi Piala Dunia 2026: Pelatih Australia Tony Popovic Tak Permasalahkan Nuansa Belanda di Timnas Indonesia	Tempo.co	20-Mar-25	https://www.tempo.co/sepakbola/kualifikasi-piala-dunia-2026-pelatih-australia-tony-popovic-tak-permasalahkan-nuansa-belanda-di-timnas-indonesia-1221890
6	Pelatih Australia Blak-blakan Soal Naturalisasi di Timnas Indonesia	CNN Indonesia	19-Mar-25	https://www.cnnindonesia.com/olahraga/20250319153143-142-1210732/pelatih-australia-blak-blakan-soal-naturalisasi-di-timnas-indonesia
7	Profil 3 Calon Pemain Naturalisasi Timnas Indonesia, Ada yang Kelahiran Mataram	Tempo.co	15-Mar-25	https://www.tempo.co/sepakbola/profil-3-calon-pemain-naturalisasi-timnas-indonesia-ada-yang-kelahiran-mataram–1211763
8	Indonesia Naturalisasi 3 Pemain Baru, Kena Sindir Pelatih Bahrain	Detik.com	12-Mar-25	https://sport.detik.com/sepakbola/liga-indonesia/d-7819688/indonesia-naturalisasi-3-pemain-baru-kena-sindir-pelatih-bahrain
9	3 Pemain Naturalisasi Anyar Perkuat Timnas Indonesia, Ini Statistiknya	Detik.com	11-Mar-25	https://www.detik.com/bali/sepakbola/d-7816939/3-pemain-naturalisasi-anyar-perkuat-timnas-indonesia-ini-statistiknya
10	Calon Pemain Timnas Emil Audero, Dean James, dan Joey Pelupessy Resmi Jadi WNI	Tempo.co	11-Mar-25	https://www.tempo.co/hukum/calon-pemain-timnas-emil-audero-dean-james-dan-joey-pelupessy-resmi-jadi-wni-1217853
11	Media Korea: Indonesia Menggila, Naturalisasi Pemain dan Tambah Power	CNN Indonesia	11-Mar-25	https://www.cnnindonesia.com/olahraga/20250311143812-142-1207527/media-korea-indonesia-menggila-naturalisasi-pemain-dan-tambah-power
12	Media Vietnam Soroti 3 Pemain Naturalisasi Baru Timnas Indonesia	CNN Indonesia	11-Mar-25	https://www.cnnindonesia.com/olahraga/20250311074102-142-1207293/media-vietnam-soroti-3-pemain-naturalisasi-baru-timnas-indonesia
13	Statistik 3 Pemain Naturalisasi Anyar RI di Eropa, Jam Terbang Tinggi!	Detik.com	11-Mar-25	https://sport.detik.com/sepakbola/liga-indonesia/d-7816897/statistik-3-pemain-naturalisasi-anyar-ri-di-eropa-jam-terbang-tinggi
14	Profil Emil Audero, Joey Pelupessy, dan Dean James, Tiga Pemain Naturalisasi Timnas Indonesia	Kompas.com	10-Mar-25	https://www.kompas.com/tren/read/2025/03/10/123000065/profil-emil-audero-joey-pelupessy-dan-dean-james-tiga-pemain-naturalisasi?page=all
15	Polemik Pernyataan Ahmad Dhani soal Naturalisasi Dianggap Cermin Kegagalan Pemilu	Tempo.co	09-Mar-25	https://www.tempo.co/politik/polemik-pernyataan-ahmad-dhani-soal-naturalisasi-dianggap-cermin-kegagalan-pemilu-1217292
16	Deretan Naturalisasi Pemain Timnas yang Disetujui sejak DPR Periode 2024	Kompas.com	06-Mar-25	https://nasional.kompas.com/read/2025/03/06/09245341/deretan-naturalisasi-pemain-timnas-yang-disetujui-sejak-dpr-periode-2024?page=all
17	Usulan Ahmad Dhani soal Naturalisasi Pemain Bola Dinilai Merendahkan Martabat Perempuan	Tempo.co	06-Mar-25	https://www.tempo.co/politik/usulan-ahmad-dhani-soal-naturalisasi-pemain-bola-dinilai-merendahkan-martabat-perempuan–1216164
18	Daftar Pemain Naturalisasi Timnas Indonesia yang Masih Aktif	CNN Indonesia	25-Feb-25	https://www.cnnindonesia.com/olahraga/20250225143332-142-1202279/daftar-pemain-naturalisasi-timnas-indonesia-yang-masih-aktif
19	Tiga Pemain Naturalisasi Baru Timnas Indonesia Diusahakan Main pada Ronde Laga Maret	Kompas.com	22-Feb-25	https://bola.kompas.com/read/2025/02/22/18191928/tiga-pemain-naturalisasi-baru-timnas-indonesia-diusahakan-main-pada-ronde-laga
20	Eks Waketum PSSI Ungkap Naturalisasi Pemain Timnas Masa Lalu untuk Tarik Minat Penonton	Republika	20-Feb-25	https://news.republika.co.id/berita/sq6llv348/eks-waketum-pssi-ungkap-naturalisasi-pemain-timnas-masa-lalu-untuk-tarik-minat-penonton
21	DPR Kritik Kebijakan Naturalisasi Pemain Timnas Indonesia oleh PSSI: Selalu Tergesa-gesa	Tempo.co	10-Feb-25	https://www.tempo.co/sepakbola/dpr-kritik-kebijakan-naturalisasi-pemain-timnas-indonesia-oleh-pssi-selalu-tergesa-gesa-1202279
22	Di Hadapan Menpora, Menkum Jamin Dukung Kebijakan Naturalisasi Pemain Timnas	Detik.com	24-Jan-25	https://news.detik.com/berita/d-7747737/di-hadapan-menpora-menkum-jamin-dukung-kebijakan-naturalisasi-pemain-timnas
23	Daftar Debut Pemain Naturalisasi Timnas Indonesia Sepanjang 2024	Tempo.co	10-Jan-25	https://www.tempo.co/sepakbola/daftar-debut-pemain-naturalisasi-timnas-indonesia-sepanjang-2024-1187913
24	Menkum Proses Permohonan Naturalisasi Satu Pemain Sepak Bola, Siapa?	Republika	10-Jan-25	https://news.republika.co.id/berita/sppx9f487/menkum-proses-permohonan-naturalisasi-satu-pemain-sepak-bola-siapa
25	Polemik Naturalisasi dan Prestasi Timnas Indonesia	Detik.com	02-Dec-24	https://news.detik.com/kolom/d-7666897/polemik-naturalisasi-dan-prestasi-timnas-indonesia
26	Profil 15 Pemain Naturalisasi Indonesia Era Shin Tae-yong	DetikSport	20-Nov-24	https://sport.detik.com/sepakbola/gila-bola/d-7591801/profil-15-pemain-naturalisasi-indonesia-era-shin-tae-yong
27	Erick Salaman dengan Tim Geypens dan Dion Markx, Isyarat Naturalisasi Pemain Berikutnya	Republika	15-Nov-24	https://news.republika.co.id/berita/smyplk348/erick-salaman-dengan-tim-geypens-dan-dion-markx-isyarat-naturalisasi-pemain-berikutnya
28	Evans Dimas Berharap Pemain Naturalisasi Timnas Indonesia tidak Dibedakan	Republika.co.id	13-Nov-24	https://news.republika.co.id/berita/smvoby348/evans-dimas-berharap-pemain-naturalisasi-timnas-indonesia-tidak-dibedakan?
29	Akmal Sebut Naturalisasi Adalah Keniscayaan dalam Sepak Bola	CNN Indonesia	05-Nov-24	https://www.cnnindonesia.com/olahraga/20241105195854-142-1163350/akmal-sebut-naturalisasi-adalah-keniscayaan-dalam-sepak-bola
30	DPR Setujui Naturalisasi Kevin Diks, Noa Johanna, dan Estella Raquel	Kompas.com	05-Nov-24	https://nasional.kompas.com/read/2024/11/05/11043641/dpr-setujui-naturalisasi-kevin-diks-noa-johanna-dan-estella-raquel
31	Mayoritas Publik Dukung Kebijakan Naturalisasi, Arya Tegaskan Komitmen PSSI Benahi Sepak Bola Indonesia	PSSI.org	05-Nov-24	https://www.pssi.org/news/mayoritas-publik-dukung-kebijakan-naturalisasi-arya-tegaskan-komitmen-pssi-benahi-sepak-bola-indonesia
32	Anita Komisi X Kritik Naturalisasi Indonesia: Kita Tidak Miskin Atlet	CNN Indonesia	04-Nov-24	https://www.cnnindonesia.com/olahraga/20241104181036-142-1162950/anita-komisi-x-kritik-naturalisasi-indonesia-kita-tidak-miskin-atlet
33	Siapa Pemain Naturalisasi Pertama di Timnas Sepak Bola Indonesia?	Tempo.co	15-Oct-24	https://www.tempo.co/sepakbola/siapa-pemain-naturalisasi-pertama-di-timnas-sepak-bola-indonesia–8994
34	Anggota DPR Tak Bangga Timnas Indonesia Naturalisasi: Bukan “Akamsi'	CNN Indonesia	19-Sep-24	https://www.cnnindonesia.com/olahraga/20240919075114-142-1145869/anggota-dpr-tak-bangga-timnas-indonesia-naturalisasi-bukan-akamsi
35	Erick Thohir: Hilgers dan Eliano Sangat Indonesia	CNN Indonesia	19-Sep-24	https://www.cnnindonesia.com/olahraga/20240919104143-142-1145937/erick-thohir-hilgers-dan-eliano-sangat-indonesia
36	Jawaban Erick Thohir soal Sampai Kapan PSSI Naturalisasi Pemain	CNN Indonesia	19-Sep-24	https://www.cnnindonesia.com/olahraga/20240919102925-142-1145930/jawaban-erick-thohir-soal-sampai-kapan-pssi-naturalisasi-pemain
37	Eliano: Saya Akan Bantu Timnas Indonesia Lolos ke Piala Dunia	CNN Indonesia	17-Sep-24	https://www.cnnindonesia.com/olahraga/20240917191557-142-1145370/eliano-saya-akan-bantu-timnas-indonesia-lolos-ke-piala-dunia
38	Menpora Bantah Pemain Naturalisasi Timnas Indonesia Pegang Dua Paspor	CNN Indonesia	17-Sep-24	https://www.cnnindonesia.com/olahraga/20240917145620-142-1145230/menpora-bantah-pemain-naturalisasi-timnas-indonesia-pegang-dua-paspor
39	Naturalisasi Hilgers dan Eliano Disetujui Komisi X, Lanjut Paripurna	CNN Indonesia	17-Sep-24	https://www.cnnindonesia.com/olahraga/20240917150430-142-1145242/naturalisasi-hilgers-dan-eliano-disetujui-komisi-x-lanjut-paripurna
40	Warna Timnas Indonesia: Mengenal Naturalisasi, Diaspora, dan Blijvers	Kompas.com	16-Sep-24	https://bola.kompas.com/read/2024/09/16/16302578/warna-timnas-indonesia-mengenal-naturalisasi-diaspora-dan-blijvers?page=all
41	Kontroversi Naturalisasi Pemain Timnas Sepak Bola yang Belum Khatam	Republika	15-Sep-24	https://kakibukit.republika.co.id/posts/330920/kontroversi-naturalisasi-pemain-timnas-sepak-bola-yang-belum-khatam-pg
42	Pengamat Sebut Banyak Pemain Naturalisasi Indonesia Punya Paspor Ganda, Ini Penjelasannya	Kompas.com	13-Sep-24	https://www.kompas.com/tren/read/2024/09/13/164500765/pengamat-sebut-banyak-pemain-naturalisasi-indonesia-punya-paspor-ganda-ini?page=all
43	Daftar 14 Pemain Naturalisasi Timnas Indonesia Era Shin Tae-yong	Kompas.com	05-Jun-24	https://www.kompas.com/tren/read/2024/06/05/140000665/daftar-14-pemain-naturalisasi-timnas-indonesia-era-shin-tae-yong?page=all
44	Daftar 11 Pemain Naturalisasi Timnas Indonesia Era Shin Tae Yong	CNN Indonesia	17-May-24	https://www.cnnindonesia.com/olahraga/20240501095558-142-1092551/daftar-11-pemain-naturalisasi-timnas-indonesia-era-shin-tae-yong
45	Geber Naturalisasi Timnas Indonesia Sudah, Modernisasi Liga 1 Kapan?	CNN Indonesia	15-Mar-24	https://www.cnnindonesia.com/olahraga/20240315163132-142-1074819/geber-naturalisasi-timnas-indonesia-sudah-modernisasi-liga-1-kapan
46	Banyak Pemain Naturalisasi, Timnas Indonesia Disindir Sebagai Timnas Belanda oleh Vietnam	Republika.co.id	13-Mar-24	https://sport.republika.co.id/berita/sa9v54456/banyak-pemain-naturalisasi-timnas-indonesia-disindir-sebagai-timnas-belanda-oleh-vietnam?
47	Edy Rahmayadi Beri Pesan untuk PSSI soal Naturalisasi Timnas	CNN Indonesia	15-Feb-24	https://www.cnnindonesia.com/olahraga/20240215142912-142-1063026/edy-rahmayadi-beri-pesan-untuk-pssi-soal-naturalisasi-timnas
48	Eks Naturalisasi Timnas Ingatkan PSSI Jangan Lupakan Talenta Lokal	CNN Indonesia	14-Jan-24	https://www.cnnindonesia.com/olahraga/20250114145600-142-1187033/eks-naturalisasi-timnas-ingatkan-pssi-jangan-lupakan-talenta-lokal
49	Media Vietnam Soroti Naturalisasi Timnas: Kualitas Indonesia Meningkat	CNN Indonesia	08-Dec-23	https://www.cnnindonesia.com/olahraga/20231208135324-142-1034654/media-vietnam-soroti-naturalisasi-timnas-kualitas-indonesia-meningkat
50	Diserang Hoaks Soal 150 Pemain Naturalisasi, Erick: Fitnah yang tidak Masuk Akal	PSSI.org	18-Nov-23	https://www.pssi.org/news/diserang-hoaks-soal-150-pemain-naturalisasi-erick-fitnah-yang-tidak-masuk-akal
51	Naturalisasi Itu Jalan Terakhir, Utamakan Bakat Sendiri	PSSI.org	20-Mar-23	https://www.pssi.org/news/naturalisasi-itu-jalan-terakhir-utamakan-bakat-sendiri
52	PSSI, Kok Naturalisasi Terus?	DetikSport	04-Nov-22	https://sport.detik.com/sepakbola/liga-indonesia/d-7622269/pssi-kok-naturalisasi-terus

### 3.2 Codification and analytical strategy

The coding process followed a three-stage thematic content analysis (22):

• Open Coding**:** Initial concepts were identified from the text (e.g., “pride”, “foreignness”, “loyalty”, “threat to locals”).
• Axial Coding**:** Relationships were established between codes and categories (e.g., naturalization as “strategy” vs. “symbolic threat”).
• Selective Coding: Emergent themes were synthesized into interpretative categories, such as *media framing of authenticity*, *national pride narratives*, and *inclusion-exclusion dialectics*.

## 4 Results and discussion

### 4.1 Naturalization as a contribution to national pride and competitiveness

The integration of naturalized players into Indonesian football has emerged as a pivotal strategy to enhance the national team's competitiveness on the international stage. Media outlets frequently highlight the positive impacts of this policy, particularly in strengthening Indonesia's national team (*Timnas Indonesia*) with players who bring experience and technical prowess from foreign leagues (23). The inclusion of these players has enabled Indonesia to be more competitive against formidable opponents in regional competitions such as the AFF Cup and Asian Cup qualifiers. The Indonesian Football Association (*PSSI*) views naturalization as a pragmatic approach to rapidly elevate the national team's quality without solely relying on the long-term development processes of local academies (24).

In recent years, several naturalized players have made significant contributions to *Timnas Indonesia*. A notable example is Stefano Lilipaly, who played a crucial role in leading Indonesia to the AFF Cup final in 2016 with his impressive performances. Lilipaly's impact demonstrates that naturalized players can become integral components of the national team's strategic framework, rather than merely filling roster spots (25). Additionally, players such as Marc Klok and Jordi Amat have infused valuable experience, particularly in leadership and defensive tactics, addressing previous vulnerabilities within *Timnas Indonesia* (26).

“Sekarang, antusiasmenya terasa begitu besar dan perasaan saya baik. Semua orang membantu saya,” harapnya. “Jadi, mari kita bakar semangat di dalam tim. Ini pertama kali saya pergi jauh, jadi saya harap dapat berbuah hasil positif melawan Bahrain demi semua orang,”. — Marc Klok in an interview on CNN Indonesia, March 24, 2025 https://posaceh.com/pemain-baru-naturalisasi-joey-pelupessy-bangga-dapat-bantu-timnas-siap-lumat-bahrain/

Beyond the technical aspects, naturalization offers psychological and motivational benefits to the national team. The presence of players with careers in Europe or other professional leagues introduces a heightened competitive mentality that can positively influence local players. This shift is evident under the guidance of coach Shin Tae-yong, where naturalized players have contributed to enhancing tactical discipline and work ethic within the team (27). By integrating players with high-level experience, local talents have the opportunity to learn and adapt to more competitive football standards. However, despite the undeniable contributions of naturalized players to the national team's performance, debates persist regarding the long-term impact of this policy. Some argue that an overreliance on naturalized players could diminish national identity and pride. Media outlets such as *Kompas* and *Republika* have highlighted the diverse public opinions on naturalized players—some view them as saviors, while others feel they overshadow local players (23).

Internationally, the practice of naturalization is not uncommon. Qatar, for instance, has adopted a similar approach by naturalizing players from South America and Africa to strengthen its national team. This strategy proved successful with their victory in the 2019 Asian Cup but also attracted criticism for potentially not reflecting the development of domestic football in Qatar (28). Indonesia can learn from this experience to ensure that naturalization does not become a short-term solution that hinders the long-term development of local players. Conversely, many naturalized players have demonstrated emotional ties to Indonesia and express pride in representing the national team. Marc Klok, for example, has openly stated his desire to contribute more to Indonesian football, not only as a player but also as an inspiration for the younger generation (29). Similarly, Sandy Walsh, who has Indonesian heritage, has expressed his pride in playing for *Timnas* and aims to leverage his European experience to aid the development of Indonesian football (30).

Nevertheless, a balanced approach is essential in implementing this policy. Uncontrolled naturalization could limit opportunities for talented Indonesian youth to develop. Research by (24) indicates that an unchecked naturalization policy may impede the regeneration of local players, ultimately weakening domestic competition. Therefore, Indonesia must ensure that naturalization is applied selectively, targeting positions that genuinely require quality enhancement. Additionally, legal and administrative aspects warrant careful consideration in the naturalization process. Some players have acquired Indonesian citizenship without undergoing deep cultural adaptation, raising questions about their genuine attachment to Indonesia or merely obtaining passports to play internationally (31). highlights cases where naturalized players did not reside long in Indonesia after their playing careers ended, prompting concerns about their long-term commitment to Indonesian football.

The naturalization of football players has significantly enhanced the performance of *Timnas Indonesia* across technical, psychological, and tactical dimensions. However, this policy must be implemented cautiously to avoid hindering the development of local players. The government and *PSSI* should formulate long-term strategies ensuring that naturalization serves as a complementary solution rather than a substitute for nurturing domestic football talent. By doing so, Indonesia can strike a balance between boosting the national team's competitiveness and maintaining the sustainability of its domestic football ecosystem.

### 4.2 Media narratives on naturalization

The naturalization of football players in Indonesia has become a subject of increasing debate among the public, academics, and sports practitioners. The government and the Indonesian Football Association (PSSI) frequently employ naturalization as a strategy to improve the quality of the national team by inviting players with international careers to strengthen the Garuda squad. This policy is claimed to be a strategic step to enhance Indonesia's competitiveness at the international level (27). However, concerns have emerged regarding the extent to which naturalized players can be considered authentic representatives of Indonesian national identity.

One of the main criticisms of the naturalization policy is the weak cultural and emotional attachment of these players to Indonesia. Some naturalized players lack strong historical ties to the country, with some even setting foot in Indonesia for the first time only after acquiring citizenship. This raises fundamental questions about whether they truly embody the spirit and character of the Indonesian nation. According to (32), cultural, historical, and linguistic elements are key components in the formation of national identity, which, unfortunately, are not fully possessed by many naturalized players.

However, there is an opposing perspective that argues naturalization is part of globalization in sports. Countries such as Qatar and China have implemented similar policies to enhance their sports performance. The difference lies in how these countries manage the integration of naturalized players into their national sports structures. Some nations require long-term commitments from foreign-born players, including cultural adaptation and contributions to grassroots sports development (33). In Indonesia, naturalization policies tend to focus more on immediate results rather than long-term strategies to ensure sustainable football development (34).

“Dalam pandangan Ivankovic, Timnas Indonesia dengan kehadiran pemain-pemain naturalisasi membuat Skuad Garuda punya karakteristik Tim Eropa. Pada laga lawan Bahrain, 10 dari 11 pemain yang tampil di tim inti adalah pemain naturalisasi.” Article CNN Indonesia “Pelatih China: Timnas Indonesia Rasa Eropa” selengkapnya di sini Head Coach China, Ivankovic in an interview on CNN Indonesia, Oct 15, 2024.: https://www.cnnindonesia.com/olahraga/20241015071259-142-1155340/pelatih-china-timnas-indonesia-rasa-eropa.

There are several impacts of naturalization on local players is another major concern. Several studies indicate that the growing number of naturalized players in the national team can hinder the development of local talents, especially in gaining opportunities to play at international events. If naturalization continues to be a short-term solution, there is a risk of dependence on foreign-born players, ultimately jeopardizing the development of young Indonesian talents (26). This presents a dilemma: does naturalization genuinely contribute to the long-term development of Indonesian football, or is it merely a pragmatic solution that stifles local talent growth?.

From a legal perspective, the naturalization of football players also presents challenges. In Indonesia, naturalization is regulated under Law No. 12 of 2006 on Citizenship of the Republic of Indonesia, which requires that naturalized citizens demonstrate ties to Indonesia, either through lineage, residency, or contributions to the country (31). However, in practice, many players obtain Indonesian citizenship without a deep-rooted connection to the country. This raises questions about whether they genuinely meet the substantive requirements for citizenship or merely fulfill administrative criteria. On the other hand, it cannot be denied that many naturalized players sincerely love Indonesia and take great pride in representing the Garuda national team. One example is Stefano Lilipaly, who, since his naturalization in 2011, has shown outstanding dedication to the Indonesian national team. Lilipaly has not only made efforts to understand Indonesian culture but has also actively communicated in Bahasa Indonesia and supported various social initiatives within the country. In an interview, he stated that playing for the Indonesian national team is the greatest achievement of his career and that he feels immense pride every time he sings the national anthem before a match (29).

Marc Klok is another example of a player who truly embraces Indonesia. Since becoming an Indonesian citizen in 2020, he has frequently expressed his love for the country through social media and interviews. Klok believes that he is more than just a naturalized player—he considers himself an integral part of Indonesian society. He is also actively involved in various social programs aimed at promoting grassroots football development in Indonesia (35). Similarly, Sandy Walsh has a strong emotional connection to Indonesia due to his heritage. Since becoming an Indonesian citizen, he has often spoken about how he feels deeply connected to Indonesian culture and history and views playing for the national team as an honor. Additionally, newly naturalized players such as Emil Audero Mulyadi and Ole Romeny have demonstrated great enthusiasm for Indonesia. Emil Audero, who previously played in Italy's Serie A, has emphasized that he is proud to represent Indonesia and hopes to use his European experience to contribute to the national team's development. Meanwhile, Ole Romeny, who recently became an Indonesian citizen, has expressed in interviews that he is eager to play for the national team and sees this as an opportunity to reconnect with his cultural roots (35).

From a social perspective, the emotional involvement of these naturalized players serves as evidence that the assumption that they only join the national team for career advancement is not entirely accurate. Many of them genuinely feel a connection to Indonesia and want to contribute to the nation's football development. Therefore, naturalization policies should not be based solely on a player's technical abilities but should also take into account their emotional and social ties to the country. In the context of national pride and identity authenticity, the presence of naturalized players who sincerely love Indonesia can serve as a reinforcing factor for national values. If they truly understand and embrace Indonesian culture, they are not merely foreign players wearing the national jersey but integral parts of the nation's journey toward football excellence. Therefore, the selection of naturalized players should be conducted more selectively, considering not only their technical skills but also their commitment to Indonesia in social, cultural, and emotional aspects.

### 4.3 Skepticism towards authenticity and implications for the development of local players

In recent years, the naturalization of football players in Indonesia has become a controversial phenomenon. While the primary goal of this policy is to enhance the performance of the national team, criticism has continued to emerge, particularly regarding its impact on local players and the authenticity of national identity. Some critics question whether naturalized players can genuinely represent Indonesia or if they merely serve as a short-term solution for national team success (36).

One of the primary concerns raised by critics is that naturalization may hinder the development of local players. As the number of naturalized foreign players increases, opportunities for domestic players to participate in international competitions become more limited. A study conducted by (24) indicates that the presence of naturalized players in the national team tends to reduce playing time for young talented players, who should be given the opportunity to develop their skills at the highest level. Additionally, the naturalization policy is feared to create prolonged dependence on foreign players. When naturalization becomes the primary strategy for improving the national team's performance, local talent development may receive less attention. This could weaken Indonesia's football ecosystem, particularly in terms of youth player development. A study by (32) highlights how other countries that have relied on naturalized players have experienced stagnation in local player development, as clubs and federations prefer quick solutions over long-term investments.

“Saya setuju saja [soal strategi naturalisasi], tapi jujur kebanggaan itu bagi saya berkurang karena dari komposisi mungkin terlalu banyak yang dinaturalisasikan, bahkan hampir satu tim,”. Read article on CNN Indonesia “Anggota DPR Tak Bangga Timnas Indonesia Naturalisasi: Bukan ‘Akamsi’” member of the council of the Republic of Indonesia Mr. Nuroji in an Interview on CNN Indonesia.

link artcile: https://www.cnnindonesia.com/olahraga/20240919075114-142-1145869/anggota-dpr-tak-bangga-timnas-indonesia-naturalisasi-bukan-akamsi.

From a national identity perspective, skepticism towards naturalized players is also related to the extent to which they genuinely understand and commit to Indonesia. Criticism of naturalization often arises because many players acquire Indonesian citizenship without undergoing a deep cultural adaptation process. A study conducted by (37) reveals that some naturalized players lack basic proficiency in the Indonesian language or knowledge of national history and culture, ultimately raising questions about their attachment to the country. Another frequently raised concern is that naturalization policies do not always consider the long-term sustainability of Indonesian football. Instead of being a long-term solution, naturalization is often used as a pragmatic strategy to enhance short-term national team performance. A study by (26) asserts that without a strong system for developing local players, the presence of naturalized players will only serve as a temporary solution, failing to address the fundamental issues within Indonesian football. The implications of this policy are also evident at the club level. Indonesian football clubs tend to prioritize recruiting foreign players eligible for naturalization over developing young talents from their academies. This practice not only disadvantages local players but also hampers the long-term progression of Indonesian football. As noted by (38), clubs that rely too heavily on naturalized players often fail to establish a sustainable regeneration system, facing challenges when these foreign players retire or experience a decline in performance.

Furthermore, the media plays a crucial role in shaping public perception of naturalized players. Media narratives often portray naturalized players as saviors of Indonesian football, while giving less attention to the challenges faced by local players in securing positions in the national team. A study by (39) demonstrates that media coverage tends to emphasize the immediate benefits of naturalization policies without critically analyzing their long-term impact on domestic football development. However, naturalization is not entirely without merit. Some naturalized players have demonstrated a strong commitment to Indonesia and have genuinely attempted to integrate into society. Players such as Stefano Lilipaly, Marc Klok, and Sandy Walsh actively engage in various social and cultural activities in Indonesia and make efforts to communicate in the Indonesian language. This suggests that, in some cases, naturalization can be successful when supported by effective integration between the players and Indonesian culture (29).

Ultimately, the biggest challenge of the naturalization policy is not only selecting high-quality players but also ensuring that this approach does not undermine the development of local players. The government and the Indonesian Football Association (PSSI) must design a more balanced policy where naturalization serves as a complement rather than a primary solution. By adopting a more strategic approach, Indonesia can benefit from naturalized players without compromising the regeneration of local talent. Skepticism regarding the authenticity of naturalized players and their impact on the development of local players should not be ignored. Without a clear and long-term-oriented policy, naturalization could backfire on the national football system. Therefore, a balanced approach is needed to ensure that naturalized players are utilized effectively while still prioritizing the development of domestic talent for the sustainable growth of Indonesian football.

### 4.4 Implications for national identity construction

#### 4.4.1 Media as a mediator between globalization and local values in the naturalization of football players in Indonesia

The naturalization of football players in Indonesia is not merely a sports policy but also reflects the dynamics of interaction between globalization and local values. Media plays a crucial role in shaping public opinion regarding the acceptance of naturalized players as part of national identity. In many cases, media acts as a mediator, helping society understand and accept the presence of naturalized players as a solution to enhancing the competitiveness of the national team at the international level. However, media also frequently questions whether this policy undermines Indonesian values in football (40).

Media coverage of naturalized players in Indonesia generally follows two dominant narratives: first, supporting naturalization as a pragmatic strategy to strengthen *Timnas Indonesia*, and second, questioning the extent to which naturalization truly reflects nationalism and national identity. A study by (23) found that media outlets such as *Kompas* and *Republika* often emphasize the short-term benefits of naturalization, such as improving national team performance. Conversely, media platforms like *Tempo* and *Tirto* tend to be more critical of the policy, highlighting issues of dependence on foreign players and its impact on the development of local talents. In many cases, media actively shapes a positive image of naturalized players in the eyes of the public. Social media and online news portals frequently highlight personal aspects of naturalized players, such as their Indonesian heritage or commitment to learning the language and culture. Coverage that portrays the emotional and cultural connections of these players aims to foster a sense of closeness between naturalized players and Indonesian society, thus increasing their acceptance as part of the national identity (27). For example, media reports on Marc Klok's efforts to learn Indonesian and his active participation in social activities have enhanced his image as an integral part of *Timnas Indonesia* (30).

However, media also tends to frame naturalization as a controversial issue linked to national identity. A major criticism highlighted by media is that naturalized players often lack sufficient emotional attachment to Indonesia and are only present to play at the international level without contributing to the long-term development of Indonesian football. Some reports have indicated that several naturalized players leave Indonesia shortly after their playing careers end, raising concerns about the sustainability of this policy (24). The debate surrounding Mees Hilgers, who initially hesitated to play for *Timnas Indonesia*, became a focal point in media discussions, questioning the loyalty of naturalized players (30).

In the context of globalization, media also plays a role in comparing Indonesia's naturalization policy with those of other countries. Several reports have discussed how nations like Qatar and China use naturalization to enhance their national teams' competitiveness. However, media also emphasizes that in these countries, naturalization is implemented with long-term strategies, including development programs ensuring that naturalized players are fully integrated into the national football system (28). In Indonesia, media often critiques the naturalization approach as being pragmatic rather than strategic, as reflected in debates over whether naturalization effectively improves football quality in the long run (41).

“Indonesia yang berpenduduk 278 juta jiwa menaturalisasi pemain dari negara yang berpenduduk 28 juta. Ini perlu dievaluasi,” Interview Edy Rahmayadi Cited from Antara.

Read article CNN Indonesia “Edy Rahmayadi Beri Pesan untuk PSSI soal Naturalisasi Timnas” link: https://www.cnnindonesia.com/olahraga/20240215142912-142-1063026/edy-rahmayadi-beri-pesan-untuk-pssi-soal-naturalisasi-timnas

Moreover, media plays a role in shaping public perception of the identity of naturalized players. In some cases, media presents naturalized players as “heroes” who bring positive change to *Timnas Indonesia*. In other cases, they are framed as “outsiders” taking spots that should belong to local players. A study by (26) suggests that media coverage can influence public acceptance of naturalized players, depending on how narratives about them are framed. This distinction is evident in the differing reactions to players like Jordi Amat, who is widely accepted, compared to others who are perceived as lacking commitment to Indonesia.

It is important to note that media's role is not always neutral. In some cases, media tends to exaggerate the positive impact of naturalization without providing an in-depth analysis of its long-term consequences. On the other hand, more critical media outlets often highlight the negative aspects of naturalization without offering constructive solutions. This imbalance in reporting can affect how society understands and evaluates the naturalization policy (25). Social media also plays a significant role in shaping public opinion on the naturalization of football players. Platforms such as Twitter, Instagram, and YouTube have become arenas for discussions on whether naturalized players can truly be considered part of Indonesia's football identity. Ultimately, media plays a central role in bridging globalization and local values in the context of naturalized football players in Indonesia. As a mediator, media helps society understand the importance of naturalization in enhancing national team competitiveness but also has a responsibility to objectively critique the policy. Therefore, balanced and data-driven reporting is essential to ensure that the public can assess the naturalization policy with a broader perspective, avoiding bias or sensationalist narratives.

#### 4.4.2 The impact of media narratives on public perception

Media plays a significant role in shaping public opinion regarding the naturalization of football players in Indonesia. The way media portrays naturalized players influences how the public perceives them—whether as assets that strengthen the national team or as threats to national identity. A study by (42) indicates that media is a key player in shaping public awareness of naturalization policies, presenting a range of narratives that either support this policy or question it in the context of nationalism and fairness for local players.

In many cases, media frames naturalized players as a quick solution to improving *Timnas Indonesia*'s performance. This narrative is particularly prevalent in sports media, which emphasizes how players like Stefano Lilipaly, Marc Klok, and Jordi Amat have contributed to enhancing the national team's gameplay. Outlets such as *Bolasport* and *CNN Indonesia* frequently highlight the positive aspects of naturalization, featuring statistical data and individual achievements of these players in international competitions. However, not all media coverage presents naturalization in a favorable light. Some media sources question the authenticity of these players' citizenship and whether they truly represent Indonesia both emotionally and culturally. An analysis by (43) shows that critical media outlets like *Tirto* and *Tempo* frequently discuss national identity issues in their reporting. They raise concerns about whether naturalization is merely a pragmatic strategy without regard for the long-term development of local players.

One of the most discussed aspects in media coverage is the cultural attachment of naturalized players to Indonesia. Players such as Sandy Walsh and Shayne Pattynama, who have Indonesian ancestry, receive more positive coverage compared to those without familial ties to Indonesia. This suggests that media plays a role in constructing narratives about who is considered “part of the nation” and who is merely playing for *Timnas* for career advancement (44).

The impact of media narratives on the public is also evident in discussions that emerge on social media. Public sentiment toward naturalized players is often influenced by how the media frames them. A study by (24) found that positive public opinions toward naturalization tend to follow media reports that emphasize the contributions of naturalized players to the national team. Conversely, skepticism rises when media focus on national identity issues and the negative impact of naturalization on local players. Media outlets also adopt different perspectives when assessing naturalization. Nationalist-leaning media often frame naturalization as a threat to the sustainability of Indonesian football, while globalist media view it as part of the broader phenomenon of globalization in sports. A study by (45) suggests that media tend to tailor their reporting to their primary audience, leading to differing perceptions among different segments of society.

Furthermore, media coverage impacts how naturalized players are received by the public. Players who receive positive media coverage tend to be more widely accepted by supporters, while those who are framed negatively often face criticism, both on social media and in stadiums. A notable example is Marc Klok, who initially faced mixed reactions upon his naturalization. However, after demonstrating strong commitment and impressive performances, he gradually gained acceptance among Indonesian football. The framing of naturalization in media also influences government policies regarding the regulation of naturalized players. When media highlights the positive effects of naturalization, the government and *PSSI* tend to be more proactive in selecting new players for naturalization. However, when media emphasizes national identity concerns and the potential disadvantages for local players, discussions about tightening naturalization policies begin to surface in public discourse and government policymaking (46). In some cases, media also plays a role in changing the perspectives of naturalized players toward Indonesia. Players who initially saw Indonesia as merely a place to play football begin to develop a deeper understanding of the country's culture and history through media exposure. This phenomenon is evident in how some players started learning the Indonesian language and engaging in social activities in Indonesia after receiving significant media attention (47). Social media platforms also serve as a crucial arena for shaping public perceptions of naturalized players. Discussions about naturalization are often driven by trending topics on platforms such as Twitter, Instagram, and YouTube. Digital campaigns that either support or oppose naturalization can quickly shape public opinion, particularly among younger supporters who are more active on social media.

In conclusion, media plays a pivotal role in shaping public perceptions of naturalized players. Through diverse narratives, media can either foster greater acceptance of naturalized players or reinforce skepticism toward this policy. Therefore, it is crucial for media to provide balanced and data-driven reporting so that the public can assess naturalization policies from a broader perspective, free from excessive bias or framing.

## 5 Conclusion & limitations

### 5.1 Conclusion

This study highlights the critical role of media narratives in shaping public perception regarding the naturalization of football players in Indonesia. This research identifies how Indonesian media construct and negotiate national identity within the broader framework of globalization. The findings demonstrate that media narratives oscillate between celebrating naturalized players as key contributors to national success and questioning their authenticity within the cultural and sporting domains of Indonesia.

The study reveals two dominant media frames: one that supports naturalization as a pragmatic strategy to enhance the competitiveness of *Timnas Indonesia*, and another that critically examines its implications for national identity and local talent development. Positive framing tends to emphasize the contributions of naturalized players, highlighting their international experience and their potential to elevate Indonesian football. Conversely, critical narratives raise concerns about overreliance on foreign-born players, the marginalization of local talent, and the authenticity of national representation.

Moreover, media discourse plays a pivotal role in influencing public sentiment and shaping policy decisions. Favorable media coverage fosters greater public acceptance of naturalized players, reinforcing their integration into the national team. On the other hand, critical narratives can amplify skepticism and drive policy discussions on tightening naturalization regulations. Additionally, social media platforms have emerged as key spaces where public debates on naturalization unfold, often mirroring or amplifying mainstream media narratives.

From a broader perspective, this study contributes to the discourse on sports, media, and nationalism by providing a non-Western case study of naturalization in football. It underscores the significance of media as a mediator between globalization and local values, demonstrating how national identity is continuously constructed and contested through sports discourse. This research underscores the profound impact of media on shaping national identity through sports, highlighting how media narratives both reflect and construct societal attitudes towards naturalization. As globalization continues to reshape national sporting identities, the role of media in mediating these transformations remains crucial. Future research should continue to explore the intersection of media, sports, and nationalism, particularly in the context of non-Western nations, where globalization and local identity often intersect in complex and contested ways.

### 5.2 Limitations and future research directions

Despite its contributions, this study has several limitations that should be acknowledged. **First**, the research primarily relies on qualitative content analysis, which, while valuable for identifying dominant themes and narratives, may be limited in capturing the full spectrum of public sentiment. Future studies could complement qualitative analysis with quantitative approaches, such as sentiment analysis or surveys, to provide a more comprehensive understanding of public opinion on naturalization. **Second**, the study focuses on major Indonesian media outlets, including *Kompas*, *Tempo*, *CNN Indonesia*, and *DetikSport*. While these sources provide valuable insights into mainstream media narratives, they may not fully represent alternative or independent media perspectives. Examining a broader range of media sources, including regional newspapers and digital-native platforms, could yield a more nuanced analysis. **Third**, this study primarily examines media narratives during naturalization. However, public discourse on naturalization is dynamic and evolves over time. Future research could adopt a longitudinal approach to analyze how media narratives shift in response to changes in government policies, team performance, or broader societal attitudes towards globalization. **Third**, while this study acknowledges the role of social media in shaping public opinion, it does not conduct an in-depth analysis of user-generated content on platforms such as Twitter, Instagram, and YouTube. Given the increasing influence of digital media in sports discourse, future studies should explore how social media users construct their own narratives around naturalized players and how these narratives interact with mainstream media coverage. **Lastly**, the study is limited to the Indonesian context, which, while valuable for understanding how non-Western nations engage with naturalization in sports, may not be directly generalizable to other countries. Comparative research examining media narratives on naturalization in different cultural and political contexts—such as Qatar, China, or European nations—could provide deeper insights into the global dimensions of sports naturalization.

## Data Availability

The data are available from the authors upon reasonable request and with the permission of the Hasanuddin University (Indonesia).
